# Physico-chemical characterization and transcriptome analysis of 5-methyltryptophan resistant lines in rice

**DOI:** 10.1371/journal.pone.0222262

**Published:** 2019-09-18

**Authors:** Franz Marielle Nogoy, Yu Jin Jung, Kwon-Kyoo Kang, Yong-Gu Cho

**Affiliations:** 1 Department of Crop Science, Chungbuk National University, Cheongju, Korea; 2 Department of Horticulture, Hankyong National University, Ansung, Korea; Murdoch University, AUSTRALIA

## Abstract

Mutation breeding has brought significant contributions to the development of high value crops. It steered the first studies to generate plants with desired mutations of genes encoding key enzymes involved in important metabolic pathways. Molecular characterization of 5-methyl tryptophan (5-MT) resistant plants has revealed different base changes in alpha unit of anthranilate synthase (*OsASA)* gene that can lead to insensitivity to feedback inhibition of anthranilate synthase. The objective of this study was to perform *in silico* analysis of microarray data from five progressing time points during grain filling of rice. Results showed various differentially expressed genes. Enrichment of these genes revealed their roles in amino acid transportation during grain filling. Surprisingly, among all DEGs, only LOC_Os06g42560, a tryptophan synthase beta chain, was found to be directly related to tryptophan biosynthesis. It might affect amino acid content during grain filling. For physico-chemical analysis, different grain and eating qualities parameters were measured using mutant rice lines. Evaluation results showed that 5MT resistant-lines (5MT R-lines) showed approximately 60% chalkiness after milling although it had 20 times higher tryptophan content measured in μg/100 mg seeds. Taste quality of these 5MT R-lines in general was not affected significantly. However, other parameters such as peak time of viscosity and gelatinization temperature showed different results compared to the wildtype. Mutant lines generated in this study are important resources for high tryptophan content, although they have lower grain quality than the wildtype. They might be useful for developing new high nutrient rice varieties.

## Introduction

Rice is the most essential staple food for about half of the world’s population. It supplies 20% of calories consumed worldwide. Rice consumption increases with increasing population. In the 21st century, a significant increase in population is anticipated. Most of the growth will occur in Asia and Africa where rice is commonly consumed [1]. With increasing yield in rice production, nutrient content of rice should also increase, especially for poor countries that rely heavily on rice. One limiting nutrient in most crops is tryptophan (Trp).

Trp is one of eight essential amino acids that our body needs but cannot synthesize. Thus, Trp must be supplied through diet. Dietary Trp is delivered to the liver. Trp is used for protein synthesis and other functions by cells throughout the body. The remaining amount of Trp not used for protein synthesis will go to other basic metabolic pathways [2]. It can be degraded in the liver through a series of metabolic steps known collectively as the kynurenine pathway [2]. In the nervous system and gut, Trp is needed as a substrate to synthesize serotonin, a brain neurotransmitter, platelet clotting factor, and neurohormone found in organs throughout the body. In the pineal gland, Trp is required to produce melatonin. When niacin content in the diet is insufficient for metabolic requirements, Trp is needed for the synthesis of nicotinamide adenine dinucleotide (NAD+), an essential cellular cofactor [2]. Collectively, Trp is needed for the production of bioactive metabolites and regulation of serotonin and melatonin. Lack of Trp can also increase the risk of pellagra disease. However, Trp supplement is expensive because of relatively low efficiency of its industrial production [3]. Trp fortification in rice for human food can contribute to great health benefits.

Anthranilate synthase (AS) can convert chorismate to anthranilate, the first reaction in Trp biosynthesis [4]. AS have alpha and beta subunits. Its alpha subunit is important for feedback inhibition of the enzyme by Trp. Thus, AS has a key role in regulating Trp biosynthesis [4]. Previous studies have characterized resistance of different crops to various analogs of Trp, including *Datura innoxia* [5], *Arabidopsis* [6], *Zea mays* [7], *Lemna gibba* [8], *Astragalus sinicus* [9], *Nicotiana tabacum* [10], and *Vigna angularis* [11]. Mutants and transgenic rice with elevated Trp content have also been characterized extensively [4, 12–16]. 5-Methyltryptophan (5MT) can fit into an allosteric site of AS, the key feedback player in the Trp biosynthetic branch of the shikimate pathway, in the same way as Trp [17]. Even a very sensitive enzyme may fail to completely differentiate between Trp and 5MT. The cell can respond to 5MT by inhibiting further Trp synthesis. However, 5MT cannot replace Trp in cellular protein synthesis, thus arresting cellular growth. Trp over-synthesizing mutants are known to be resistant to growth inhibition [17].

For this study, two 5MT resistant-lines (5MT R-lines) were characterized by observing their *in vitro* growth inhibition, confirming point mutations in AS gene, testing AS enzyme activity, quantifying mRNA expression of genes encoding the enzymes involved in Trp biosynthetic pathway and amino acid content analysis in grains. Analysis of microarray data collected from progressing time points during grain filling of selected 5MT R-lines was performed to determine the amino acid transporters responsible for delivering Trp in mature grains of mutant rice lines.

## Materials and methods

### Plant materials and growth conditions

For characterization, plant materials were limited to two mutant lines derived from seeds mutated through 2% ethyl methanesulfonate (EMS, CH_3_SO_3_C_2_H_5_). These two mutant lines, 5MT-4 and 5MT-5, have undergone several generations of selfing. Thus, they are considered as uniform and stable mutant lines. The wildtype (WT) rice variety is Donganbyeo.

Dehulled mature seeds of WT, 5MT-4, and 5MT-5 were surfaced sterilized by washing once with 70% ethanol for five minutes followed by washing with 2.5% sodium hypochlorite twice (ten minutes each). Seeds were then rinsed several times with sterile distilled water. Seeds were air dried for an hour, inoculated into half strength Murashige and Skoog (MS) medium containing 50 μM 5-Methyl-DL-tryptophan (M0534 Sigma), and grown for 10 days at 30°C under constant light.

### Isolation of *OsASA* genes from mutant lines

Point mutations of anthranilate synthase gene have already been identified in a previous research project [18]. To verify mutations present in 5MT-4 and 5MT-5, the following primers flanking the previously reported point mutations were designed: 5’-GGGGAAGCCAGAGGCAAG-3’ and 5’- CCTGGGGATCTGCATAGGAT-3’ flanking the amino acid region F124V and S16F in LOC_Os03g15780. The target size was 388 base pairs. After obtaining the correct size of amplicon, direct sequencing was performed using the forward primer only. Sequence of LOC_Os03g15780 was used as reference sequence. Sequencing results of WT, 5MT-4, and 5MT-5 were aligned using online sequence alignment platform MultAlin [19]. To confirm mutation L530D in anthranilate synthase, primers 5’-TTTGCTCTGTTGACTTCGATCA-3’ and 5’-GTTCTCGCATTCACGTTGCT-3’ were used with target size of 650 bp. The PCR product was electrophoresed on 1% agarose gel to check target amplicon, subcloned to pGEMT-easy vector, and then sequenced.

### Anthranilate synthase assay

Leaves of 2-week-old seedlings grown in half strength MS vitamins with 5MT were ground with liquid nitrogen using mortar and pestle and suspended in one volume of ice-cold AS extraction buffer and 2 mg polyvinylpolypyrrolidone per 1 g of fresh leaves. The solution was centrifuged at 30,000 g for 15 minutes twice. One volume of the collected supernatant was mixed with two volumes of saturated ammonium sulphate and centrifuged. The resulting pellet was then re-dissolved in 500 μL AS extraction buffer for later use in AS assay. AS activity was measured based on chorismate-dependent production of anthranilate. Re-suspended ammonium sulphate pellet was combined with 1.5 ml of AS Buffer 2 and 200 μL of substrate solution. Anthranilate levels were quantified fluorometrically at 340 nm (excitation) and 400 nm (emission) using an Aminco-Bowman spectrofluorimeter (SLM-Aminco, Urbana, IL, USA). Anthranilate formation was subjected to continuous fluorometric monitoring at 30°C with the stepwise addition of small volumes (1, 10, 100, 250, 500 and 1000 mM) of Trp stock solution (5 mM). The reaction was allowed to stabilize between each addition.

### qRT-PCR of Trp synthesis related genes

To determine the expression of genes downstream of anthranilate synthase gene in Trp biosynthesis pathway, primers for anthranilate synthase alpha subunit 1 (*OsASA1*), anthranilate synthase alpha subunit 2 (*OsASA2*), anthranilate phosphoribosyltransferase (*OsAPT*), tryptophan synthase alpha chain (*OsTSAC*), and tryptophan synthase beta chain (*OsTSBC*) were designed (S1 Table). Leaf samples were collected from one-week-old seedlings and used for crude RNA extraction with RNAiso Plus (Total RNA extraction reagent). ReverTra Ace^®^ qPCR RT master mix with gDNA remover was used for cDNA synthesis. For qRT-PCR, iQ^™^ SYBR^®^ Green supermix and Bio-Rad real time PCR instrument were used. The house keeping gene employed here was *ubiquitin 10*. Primer sequences used in this experiment are listed in S1 Table.

### Potassium-iodide (KI) analysis of starch

Iodine-KI (I_2_KI) reagent was prepared by dissolving 1 g potassium iodide in 10 ml of distilled water followed by addition of 0.5 g iodine crystals. Each rice grain was cut in cross-section and a very small drop of I_2_KI solution was added to allow starch to react in the presence of iodine. A deep blue-black color will appear when amylose is present. If starch amylose is not present, then the color stays orange or yellow. Starch amylopectin, cellulose, or disaccharide does not give any color.

### Estimation of amylose content

The percentage of amylose content was determined using a previously published protocol [20]. Briefly, for each sample, 10 mg of powdered milled rice was mixed with 100 μl of 100% ethanol and 900 μl of 1 N NaOH. After thorough mix, samples were heated to boiling at 100 °C for 10 minutes. Test tubes were cooled, mixed on a vortex mixer, and filled to 10 ml mark with distilled water. To make a technical replicate, 500 μl of sample was added into a new 20 ml test tube containing 5 ml of distilled water. The sample was neutralized with 100 μl of 1 M acetic acid followed by addition of 200 μl of fresh iodine-potassium iodide solution (120 mM KI, 8 mM I_2_) and 4.2 ml of distilled water. After mixing well with a vortex mixer, tubes were incubated at 30 °C in a dark room for 20 mins. One ml of each sample was pipetted in a cuvette. Absorbance of each sample was measured at wavelength of 620 nm using a spectrophotometer (Shimadzu). Standard series (0%, 4%, 8%, 12%, 16% and 20%) were prepared the same way using pure potato starch as amylose. A standard curve was then made using absorbance readings.

### Amino acid content analysis

To quantify amino acid content, specifically Trp, in grains of WT, 5MT-4, and 5MT-5, one gram of ground sample of each brown and milled rice were sent for amino acid content analysis in Korea Basic Science Institute (KBSI), Daejeon, South Korea. Amino acid composition analysis system of KBSI was composed of an HPLC system (separation module), a UV/visible detector, a fluorescence detector, and a chromatography software. Samples were replicated twice. Analysis did not employ acid hydrolysis condition to avoid loss of Trp known to occur in most acidic solutions. A similar detailed protocol can be adapted for HPLC–MS/MS instrumentation, chromatographic and MS parameters [21].

### Growth and tissue collection of 5MT R-lines

For gene profiling analysis, the plant materials used were the wildtype rice (Donganbyeo) and the mutant rice (5MT R-line) with a high total amino acid, the same materials which were also used from the previous study [18]. Samples were collected from different grain filling stages at 2, 5, 10, 15, and 20 days after pollination (DAP). Grains of each DAP stage with three biologically independent replicates were rapidly frozen in liquid nitrogen and ground to powder using mortar and pestle. Total RNA was extracted from grain tissues using TAKARA RNAiso plus. Quantity and quality of RNA samples were determined using a Nanodrop ND 1000 spectrophotometer (Nanodrop Technologies Inc., Rockland, DE, USA). Using RiceXPro website, integrity of samples was verified with an Agilent 2100 Bioanalyzer (Agilent Technologies, Santa Clara, CA, USA). Total RNA was labeled with Cy3 or Cy5 using an Agilent Quick Amp Labeling kit (Agilent Technologies). Fluorescently labeled targets were hybridized to Agilent Rice 4 X 44K microarray. Hybridization and washing were performed following the manufacturer’s instructions. Hybridized microarrays were scanned and quantified using GenePix Pro 4.0. LOWESS program was used for normalization and Agilent GeneSpringGX 7.3 was used for data analysis.

There were five sets of microarray data of grain filling stages defined above. Poor quality data were called out based on their flag values. Any data with A (absent/bad) and M (marginal/intermediate) flag values were removed. All entries with *P*-values ≤ 0.05 were then selected. From this list, fold change expression was computed for each gene using the formula of log_2_ FC = log2 (mutant/wildtype). Those with ≥ 2-fold change in expressed were selected.

### Gene ontology analysis

Using the final gene list (S2 Table), various analyses were performed to determine gene distribution in each stage and overlapping gene expression (S1 Fig). To understand amino acid transporters (AATs), gene ontology analysis was performed for all up- and down-regulated differentially expressed genes (DEGs) in mutant/wildtype plants. The background organism used was always *Japonica* rice. Bioinformatics platforms used were Gene Ontology Consortium (http://geneontology.org/), PANTHER (Protein ANnotation THrough Evolutionary Relationship) classification system (http://pantherdb.org/), and DAVID (https://david.ncifcrf.gov/). Graphical plotting was prepared in Excel and R Studio.

### Physico-chemical analysis

After harvest, rice grains were dried in an oven at 65°C for three days. After ensuring that grains had moisture content of about 14%, grains were hulled with a rice huller to make brown rice. Brown rice samples were then polished to 90% by a polishing machine (MC-90A, Toyoseiki). Fifteen grams of polished rice samples were weighed and milled through a 100-mesh screen using Cyclotec Sample Mill (Foss North America (Tecator) No.1093-003). Ground samples were used for viscosity test, amylose content analysis, and amino acid content analysis.

A grain selector (Takemoto Electric, Co., Tokyo, Japan) was used to separate broken rice from whole grains. Grains selected were used for further analysis. A grain inspector machine (FOSS, Denmark) was used to measure whole grains, brokenness, chalky grains, and damaged grains. An Infratec grain analyzer (RVA; Model No. RVA-4; Newport Scientific, Warriewood, NSW, Australia) was used to measure moisture, protein, amylose, and whiteness of grains.

To measure the viscosity of rice samples in this study, a Rapid Visco Analyzer (RVA) (RVA-4 Newport science inc. Australia) was used. RVA was developed by The Food Agency in Japan. It provides a long profile to better differentiate rice samples of similar quality. A canister was filled with 25.0 ml of distilled water. Selected ground rice samples (three grams) were transferred to the canister. Into the canister. The paddle was placed properly and the canister was inserted into the RVA instrument. All viscosity parameters were measured in rapid visco units (RVU) using three replicates for each sample.

For palatability, two machines were used. The first one was a Satake Rice Taste Analyzer requiring about 10 grams of milled rice. The other machine was a TOYO taste meter system (MB-90A and MA-90B, Japan) which uses 33 g of milled rice per sample [22]. Detailed procedures of grain and eating quality evaluation used in this study were previously reported [23]. Some protocols were adapted from Rice Quality Training Manual of International Rice Research Institute.

## Results

### Growth response under 5-methyl tryptophan

Two mutant rice lines generated by EMS treatment were characterized and evaluated using molecular and phenotypic analyses. First, seeds were selected by growing in half strength MS media with 50 μM of 5MT, an analog of Trp that could inhibit the synthesis of anthranilate compounds known to be the first enzymes in the biosynthesis of Trp. WT and two mutant rice lines were both grown in half strength MS media without 5MT. All three lines showed good growth performance in the control medium. Ten days after growing in inhibition media, well-developed shoots and roots of 5MT-4 and 5MT-5 were selected. It was very obvious that roots of WT did not develop well. Various biological and technical replicates were also used for this growth test and similar results were obtained.

### Anthranilate synthase gene

**Fig 1:**
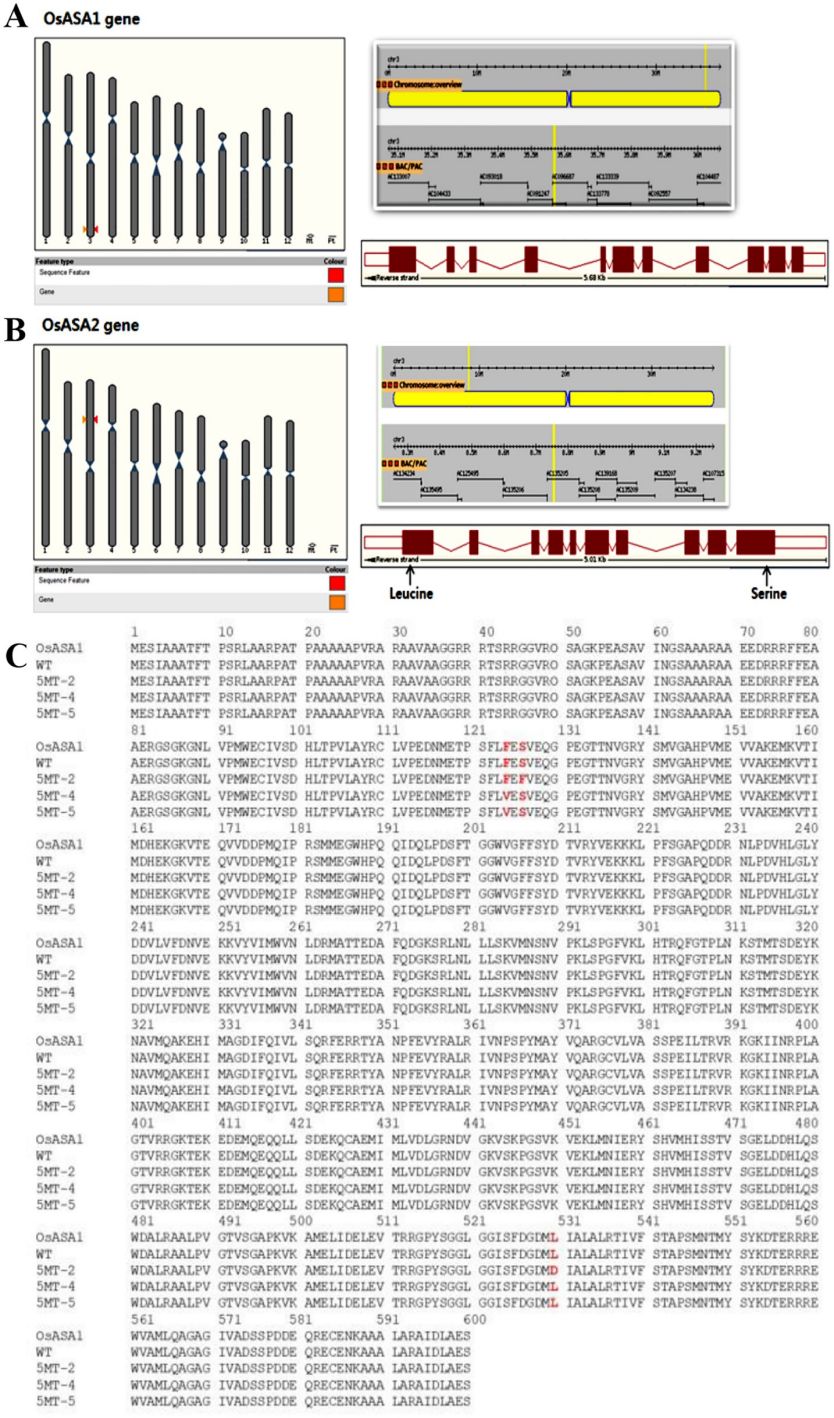
Genomic structure and transcript analysis of (A) *OsASA1* and (B) *OsASA2* genes. Small orange and red head arrows denote the location of each gene in the chromosome (C). Deduced amino acid sequence of *OsASA1* reveals different mutation sites of 5MT-2, 5MT-4, and 5MT-5. Amino acid base changes are shown in red fonts.

Anthranilate synthase is a key enzyme in the feedback inhibition of Trp synthesis. It has two subunits known as *OsASA1* (LOC_Os03g61120) and *OsASA2* (LOC_Os03g15780) [4]. *OsASA1* is located on the short arm of chromosome 3 with 11 exons with a complete open reading frame (ORF) (1,831 bp) (Fig 1A). *OsASA2* gene is located in the long arm of chromosome 3. It has 10 exon regions and 9 intron regions with an ORF of 1,821 bp (Fig 1B).

To check mutations in 5MT-4 and 5MT-5, *OsASA1* gene was sequenced via direct sequencing of PCR amplicon. Raw sequences were translated into amino acid sequences. Results confirmed a single base change in AS gene, leading to an amino acid change from phenylalanine to valine in position 124 (F124V). In addition to this single base mutation found in *OsASA1* subunit, a previous study [18] has reported two more point mutations in *OsASA2* subunit in different mutant lines other than 5MT-4 or 5MT-5. Instead of direct sequencing of PCR amplicon, the product was digested with *HindIII* to cut it into two fragments, subcloned into pGEMT-easy vector, and sequenced. The whole assembly of nucleotide and amino acid sequence of *OsASA2* of 5MT insensitive mutant rice along with point mutations TCC (S) in the 126 bp region (S126F) and CTT (L) in the 530 bp region (L530D) are shown in Fig 1C.

AS activity in different concentrations of Trp (0, 10, 100, 250, 500, 1000 μM) was measured using 10 mM glutamine and 100 μM chorismate as substrates. The result showed an increasing trend of AS from extracts of two high Trp lines when Trp concentration was increased. 5MT-5 showed 1.5 times higher activity compared to 5MT-4 at the end. In contrast, the wildtype showed low AS activity [23].

**Fig 2:**
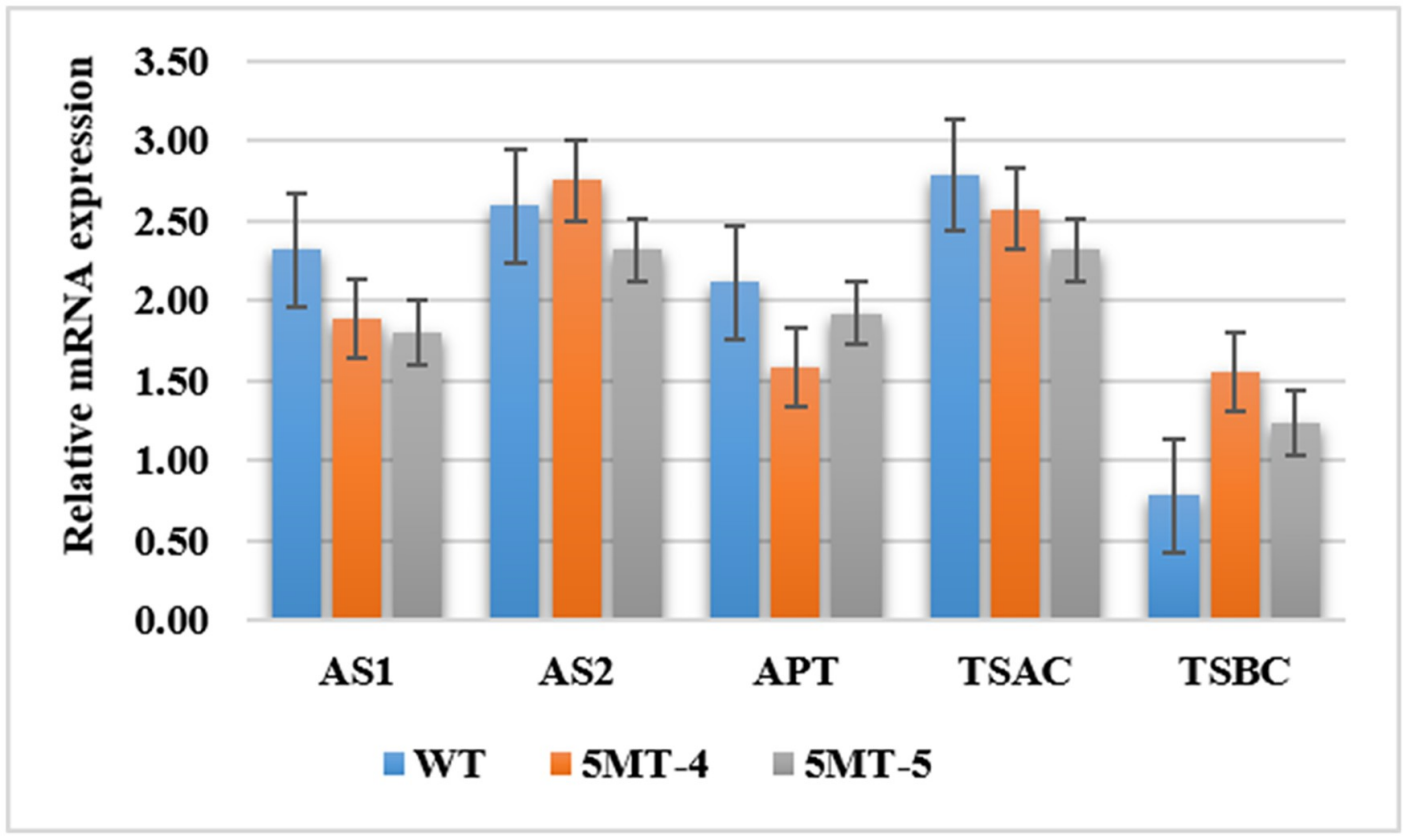
Expression of rice genes encoding enzymes in Trp biosynthesis pathway from pooled tissues of leaf, stem, and root. AS1, anthranilate synthase alpha subunit1; AS2, anthranilate synthase alpha subunit2; APT, anthranilate phosphoribosyl transferase; TSAC, tryptophan synthase alpha chain; and TSBC, tryptophan synthase beta chain.

For genes encoding enzymes downstream of the Trp biosynthesis pathway, qPCR was conducted using pooled tissues of leaf, stem, and root from each mutant and wildtype. Fig 2 clearly shows a difference in gene expression between the two 5MT R-lines and the wildtype. The trend of downstream genes APT, TSAC, and TSBC collectively in the two 5MT R-lines was similar to that in the WT. This may infer no significant changes in the synthesis pathway of Trp between the wildtype and 5MT R-lines.

### Amino acid content of 5MT R-lines

Grains of 5MT-4, 5MT-5, and WT were prepared for amino acid content analysis. Mean contents of twenty amino acids obtained from brown rice and milled rice are shown in Table 1. Duncan’s multiple range test revealed that contents of various amino acids were significantly increased in 5MT-4 and 5MT-5 than those in WT. All values of amino acid content are presented in mg/100 mg. In brown rice data, amino acids that were significantly highest among the three were aspartic acid (Asp), glutamic acid (Glu), asparagine (Asn), serine (Ser), glutamine (Gln), glycine (Gly), threonine (Thr), alanine (Ala), valine (Val), isoleucine (Ile), leucine (Leu), tryptophan (Trp), and lysine (Lys). For milled rice, there was an evident decrease in the amino acid content. However, highly significant values were still identified among the three plant materials. The same amino acids from brown rice were found Gly, Thr, Ile, Leu, and Lys. In addition to the list, histidine (His) and methionine (Met) contents were increased.

**Table 1:** Amino acid content of milled and brown rice forms of 5MT R-lines.

Amino acid	Milled rice (μg /100 mg)			Brown rice (μg /100 mg)		
	WT	5MT-4	5MT-5	WT	5MT-4	5MT-5
Asp	11.59^c^	16.95^a^	15.47^b^	17.86^c^	25.66^a^	21.39^b^
Glu	6.37^b^	9.71^a^	8.70^a^	13.27^b^	16.15^a^	10.86^c^
Asn	4.08^c^	6.54^a^	5.19^b^	6.68^b^	11.02^a^	7.72^b^
Ser	1.59^b^	2.00^a^^b^	2.42^a^	2.08^b^	2.98^a^	2.12^b^
Gln	4.93^c^	6.49^b^	7.35^a^	3.94^b^	6.31^a^	3.01^c^
Gly	2.15^a^	1.41^a^	1.48^a^	1.20^c^	1.51^b^	1.85^a^
His	2.40^b^	3.01^a^^b^	4.02^a^	3.73^a^	4.45^a^	4.14^a^
Arg	6.32^a^	6.32^a^	6.63^a^	7.30^a^	9.17^a^	8.47^a^
Thr	1.45^a^	1.92^a^	2.22^a^	1.80^b^	2.32^a^	2.54^a^
Ala	2.41^b^	3.14^a^	3.33^a^	4.78^b^	6.74^a^	5.21^b^
Pro	2.33^a^	2.30^a^	2.64^a^	2.72^a^	2.08^a^	1.85^a^
Tyr	0.00	0.00	0.00	1.57^a^	2.15^a^	2.01^a^
Val	1.11^b^	1.27^b^	1.83^a^	1.08^b^	1.69^a^	1.15^b^
Met	0.00^c^	0.63^b^	1.26^a^	0.73^a^	0.81^a^	0.74^a^
Cys2	1.94^a^	0.00^b^	0.00^b^	0.00	0.00	0.00
Ile	1.57^a^	1.70^a^	1.84^a^	1.47^b^	1.58^a^	1.38^b^
Leu	1.08^a^	1.17^a^	1.25^a^	1.08^a^^b^	1.16^a^	0.90^b^
Phe	0.00	0.00	0.00	0.00	0.00	0.00
Trp	**0.00**^c^	**6.41**^b^	**8.13**^a^	**2.39**^c^	**52.69**^b^	**66.09**^a^
Lys	0.61^a^	0.91^a^	0.80^a^	0.95^b^	1.47^a^	1.34^a^^b^
TOTAL	51.93^b^	71.86^a^	74.58^a^	74.62^c^	149.93^a^	142.78^b^

^a,b,c^ Mean with the same letter in a row are not significantly different based on Duncan grouping at α = 0.05.

Surprisingly, Cys2 was detected only in milled rice of WT. In brown or milled rice, Trp contents in 5MT-4 and 5MT-5 were significantly higher than those in WT.

### Gene expression profiling

A representative of 5MT R-line has been previously reported to have significantly higher total amino acid content (AAC) compared to its wildtype. Results of comparison of total AAC between wildtype and 5MT R-lines across five different DAPs are shown in Table 2. At 2 DAP, the wildtype had a large amount of total AAC. Total AAC then decreased drastically at 5 DAP until early maturity of the seed (20 DAP). This suggests the possible use of nutrients during grain filling. The opposite trend was found in the 5MT R-line. Total AAC decreased from 2 DAP to 5 DAP but at 20 DAP. Thus, it would be interesting to determine changes in gene expression across different time points.

**Table 2:** Amino acid content in WT and 5MT R-line at different time points of grain filling.

Rice line	Total amino acid content (μg/100 mg)				
	2 DAP	5 DAP	10 DAP	15 DAP	20 DAP*
Wildtype	1,410.2	218.3	170.7	125.5	108.3
5MT R-line	1,313.2	260.4	181.0	183.2	252.8

* DAP: days after pollination

### Functional annotation of DEGs between wildtype and 5MT R-line

**Fig 3:**
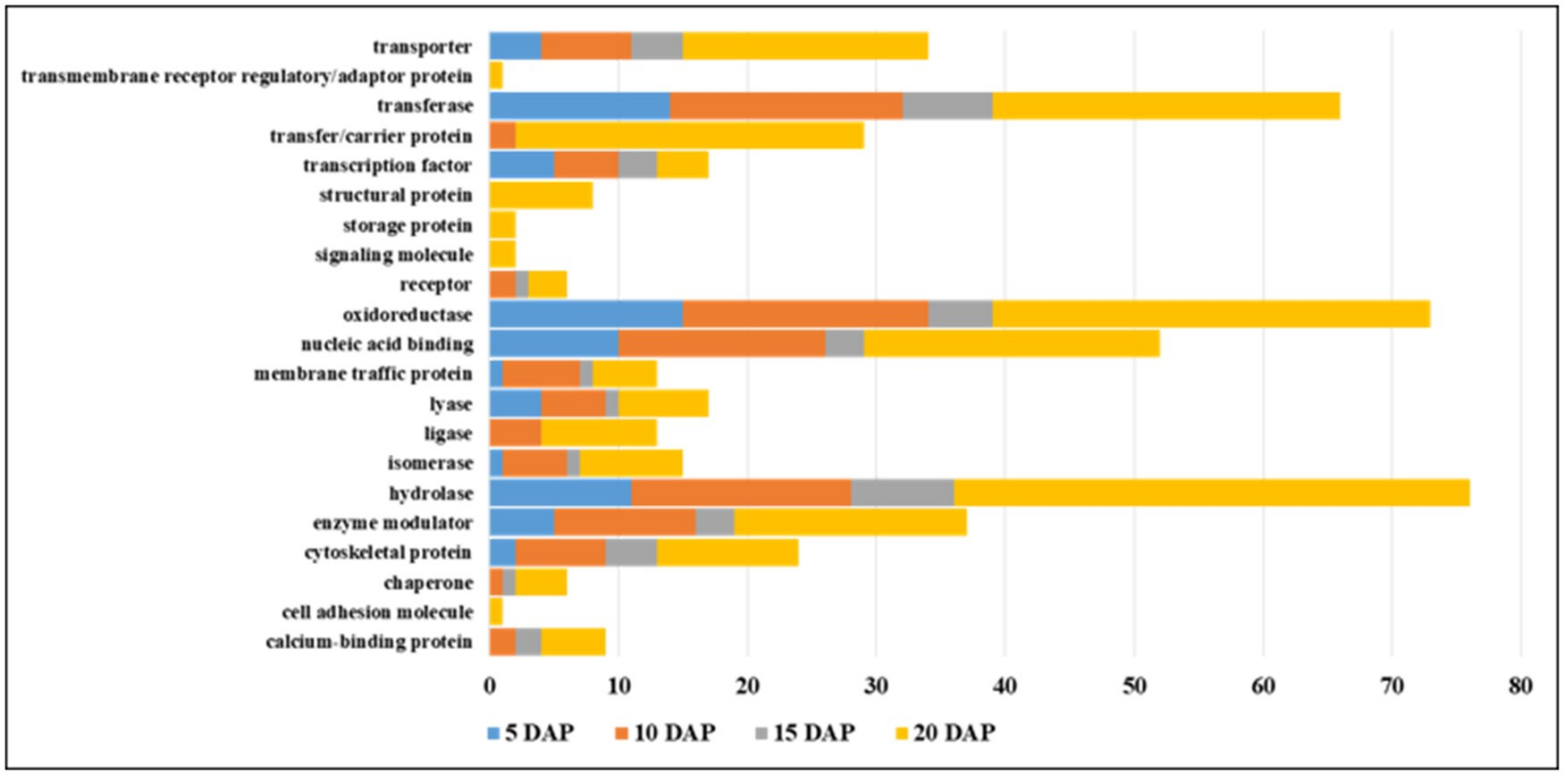
Frequency of differentially expressed genes during grain filling stage based on their protein class.

All DEGs present in 5, 10, 15, and 20 DAP were identified and classified based on their chromosome distribution (S1 Fig) and protein classification (S2 Table) in Panther database [24]. Overlapping and unique up and down regulated DEGs across the four DAP is visualized in S2 Fig. The top three DEGs classified with the largest numbers were hydrolase (n = 76), oxidoreductase (n = 73), and transferase (n = 66) (Fig 3). There were 29 DEGs associated with transfer/carrier protein class and 34 DEGs related to transporter protein class. Translocation of amino acids was also highlighted in 5MT R-line. DEGs related to transporter and transfer/carrier protein classes are listed in Table 3. Protein classes such as transmembrane receptor-regulatory, storage protein, signaling molecule, structural protein, and chaperone had the least number of DEGs. They were only present in 20 DAP.

**Table 3:** Mapped genes from Panther Gene Ontology transporter protein class.

MSU_loci	PANTHER Protein Class	David’s Functional Annotation	DAP	Up/Down
LOC_Os03g53110	cation transporter	magnesium transporter MRS2-I (LOC4334067)	10	up
LOC_Os03g19290	amino acid transporter	outer envelope pore protein 16–2, chloroplastic (LOC4332597)	20	up
LOC_Os09g12720	ion channel, metalloprotease	uncharacterized LOC4346658 (LOC4346658)	20	up
LOC_Os07g41330	mitochondrial carrier protein, transfer/carrier protein	mitochondrial import inner membrane translocase subunit TIM17-2 (LOC4343846)	20	up
LOC_Os04g36040	transporter	protein NRT1/ PTR FAMILY 4.4 (LOC4335932)	20	up
LOC_Os01g31870	cation transporter	metal transporter Nramp4 (LOC4324486)	5	up
LOC_Os02g55440	transporter	transmembrane 9 superfamily member 11 (LOC4331019)	20	up
LOC_Os07g38610	transporter	CMP-sialic acid transporter 2 (LOC4343687)	20	up
LO C_Os01g08020	transporter	Boron transporter 2	10	up
Os01g0960800 (RAP)	transporter	mitochondrial import inner membrane translocase subunit tim16 (LOC9268634)	20	up
LOC_Os12g32940	carbohydrate transporter	polyol transporter 5 (LOC4352335)	10	up
LOC_Os03g15540	transfer/carrier protein, transporter		10	up
LOC_Os01g68510	transporter	protein NRT1/ PTR FAMILY 2.13 (LOC4324322)	5	up
LOC_Os04g30740	transporter	uncharacterized LOC4335608 (LOC4335608)	10	up
LOC_Os05g01560	ATP synthase, hydrolase	V-type proton ATPase 16 kDa proteolipid subunit (LOC4337563)	20	down
LOC_Os03g11010	cation transporter	metal transporter Nramp2 (LOC4332012)	20	down
LOC_Os10g30590	RNA binding protein, serine/threonine protein kinase receptor, transporter	pentatricopeptide repeat-containing protein At5g08305 (LOC4348732)	20	down
LOC_Os07g01090	amino acid transporter	probable proline transporter 2 (LOC4342166)	20	down
LOC_Os01g17240	cation transporter	probable anion transporter 1, chloroplastic (LOC4326905)	20	down
LOC_Os01g65840	RNA binding protein, serine/threonine protein kinase receptor, transporter	pentatricopeptide repeat-containing protein At3g21470 (LOC4324845)	5	down
LOC_Os02g09810	amino acid transporter	sodium-coupled neutral amino acid transporter 1 (LOC4328577)	20	down
LOC_Os04g43070	transporter	ammonium transporter 1 member 1 (LOC4336365)	20	down
LOC_Os02g01100	amino acid transporter	vacuolar amino acid transporter 1 (LOC4327977)	20	down
LOC_Os07g18874	ATP-binding cassette (ABC) transporter	ABC transporter G family member 11 (LOC4342943)	15	down
LOC_Os03g10640	cation transporter, hydrolase, ion channel	calcium-transporting ATPase 2, plasma membrane-type (LOC4331984)	10	down
LOC_Os08g29570	ATP-binding cassette (ABC) transporter	ABC transporter G family member 44 (LOC4345450)	15	down
LOC_Os10g42830	carbohydrate transporter	D-xylose-proton symporter-like 2 (LOC4349512)	20	down
LOC_Os02g57720	amino acid transporter	aquaporin PIP 1–3 (LOC4331194)	20	down
LOC_Os07g33780	ATP-binding cassette (ABC) transporter	ABC transporter G family member 43 (LOC4343412)	15	down
LOC_Os04g38680	amino acid transporter	vacuolar amino acid transporter 1 (LOC4336049)	20	down
LOC_Os08g43120	ATP-binding cassette (ABC) transporter	ABC transporter G family member 45 (LOC4346210)	10, 15, 20	down
LOC_Os09g36930	amino acid transporter	probable aquaporin PIP2-7 (LOC4347729)	5	down

### Elucidating amino acid transporters in 5MT R-line

To elucidate amino acid transporters in 5MT R-line, the list of genes that were classified as transporter in Table 3 were extracted and annotated using Panther and David’s databases. There were 31 genes that belonged to transporter protein class. There were 14 up-regulated genes and 18 suppressed genes. Based on the classification of AATs found, there were cation transporters, mitochondrial carrier proteins, general transporters, and ATP-binding cassette (ABC) transporters. These transporters can carry different amino acids, magnesium, and even other metal forms. Although only a few DEGs related to AATs were found, these genes could play a major role in transporting amino acids from nitrogen source. More specifically, they could help break down macromolecules like sugar and carbohydrates and utilize them during grain filling.

There were five genes classified as transfer/carrier proteins. Five of them were up-regulated and one was down regulated (Table 4). Based on the Rice Genome Annotation project, gene product names of the four up-regulated genes were: LOC_Os03g15540, a putative HEAT repeat family protein; LOC_Os01g01350, gene that was upregulated in both 10 and 20 DAP, an SNF7 domain containing protein; LOC_Os07g41330, a mitochondrial import inner membrane translocase subunit Tim17; and LOC_Os08g20420, a monogalactosyl diacylglycerol synthase 2.

**Table 4:** Mapped genes from Panther Gene Ontology transfer/carrier protein class.

MSU_loci	PANTHER Protein Class	David’s Functional Annotation	DAP	Up/Down
LOC_Os03g15540	transfer/carrier protein, transporter		10	up
LOC_Os01g01350	transfer/carrier protein	charged multivesicular body protein 5 (LOC9266052)	10	up
LOC_Os07g41330	mitochondrial carrier protein, transfer/carrier protein	mitochondrial import inner membrane translocase subunit TIM17-2 (LOC4343846)	20	up
LOC_Os08g20420	acetyltransferase, glycosyltransferase, transfer/carrier protein	probable monogalactosyldiacylglycerol synthase 2, chloroplastic (LOC4345212)	20	up
LOC_Os01g01350	transfer/carrier protein		20	up
LOC_Os06g02490	enzyme modulator, transfer/carrier protein	acyl-CoA-binding protein (LOC4339921)	20	down

**Fig 4:**
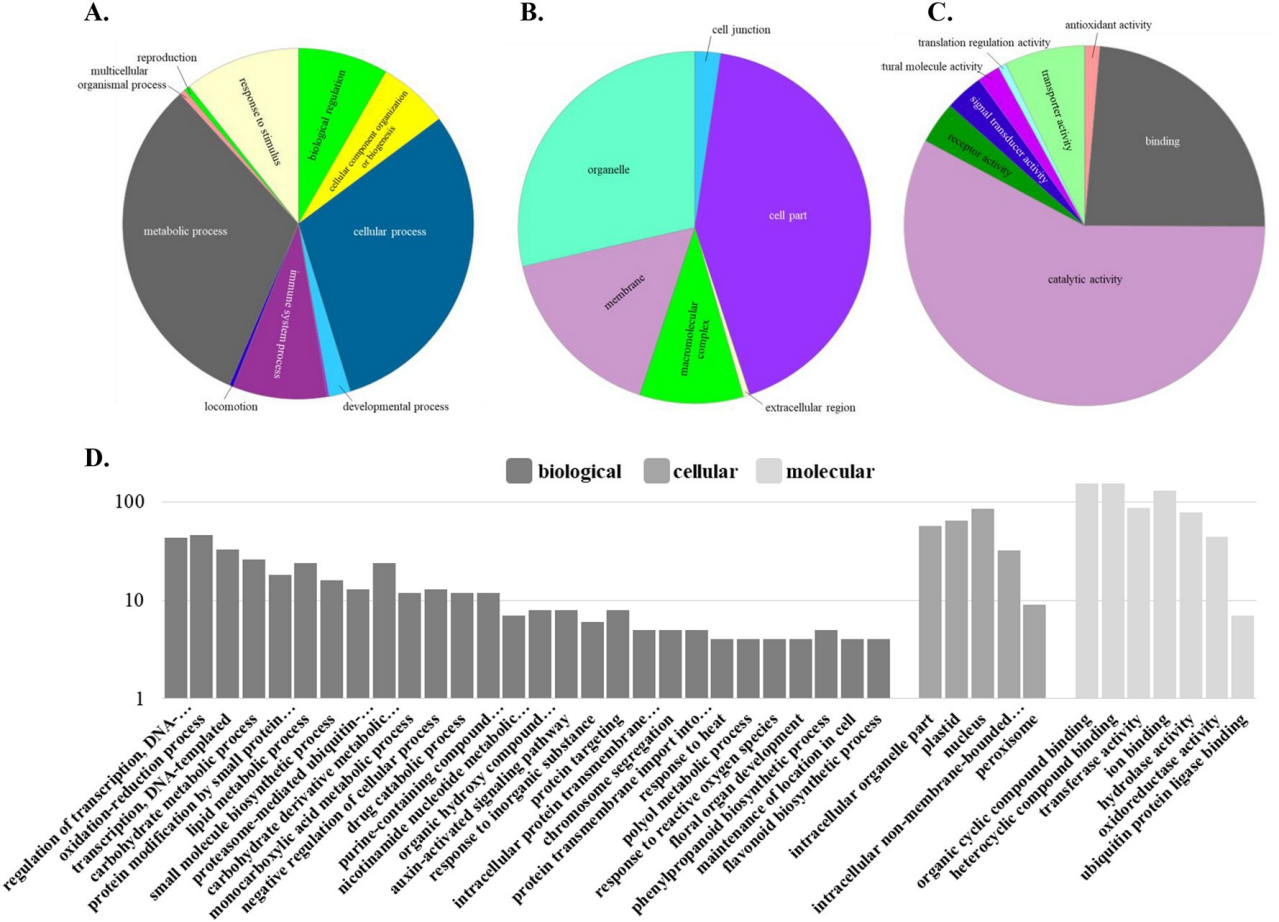
Functional categories of DEGs of mutant/wildtype at twenty days after pollination.

For functional categorization of DEGs, functions associated in each time point were first described. At 5 DAP, DEGs were assigned to three gene ontology classes: biological process, cellular component, and molecular function (Fig 4 and Table 5) using Panther Classification System [25–26]. To compare gene classifications to other bioinformatics functional platform, Gene Ontology (GO) powered by Panther [27] was used. To help us define what these classifications were based on, biological process—the larger processes, or ‘biological programs’ made by multiple molecular activities; molecular function means molecular-level activities created by gene products; and cellular component simply pertains to the locations relative to cellular structures in which a gene product does its function. At all four time points, in terms of biological processes, metabolic and cellular processes were more prominent. For the location of gene functions, most of them occurred in organelle and cell part. Regarding molecular function, catalytic activity was the most evident across 5, 10, 15, 20 DAP. In GO analysis, increasing functions can be classified as number increase of DEGs in the list. At 5 DAP, most DEGs were given classification at molecular level. At 10 DAP, functional classification became more evident in biological processes and continued at 15 and 20 DAP (Fig 4). Among all DEGs, LOC_Os06g42560, a tryptophan synthase beta chain, was the only gene found to be involved in Trp biosynthesis pathway. It showed two-fold decrease in gene expression. This proves that either alpha or beta subunit of *OsASA* can play a role in regulating the biosynthesis of Trp in rice. Further, very dynamic changes in classifications of DEGs in all four time points denoted a very dynamic processes that happened during grain filling of 5MT R-line.

**Table 5:** Functional categories of DEGs between 5MT R-line mutant rice and wildtype at 5, 10, and 15 DAP.

< Biological >	No. of DEGs		
	5 DAP	10 DAP	15 DAP
carboxylic acid biosynthetic process			9
carboxylic acid metabolic process		15	
cellular carbohydrate catabolic process	4		
cellular component organization or biogenesis		25	
cofactor metabolic process			11
intracellular transport		11	
negative regulation of endopeptidase activity			5
organonitrogen compound metabolic process			51
organophosphate metabolic process	10		
oxidation-reduction process			26
pigment metabolic process			4
primary metabolic process			75
pyrimidine nucleobase metabolic process		3	
pyrimidine nucleoside salvage		3	
response to abiotic stimulus		9	
response to chemical		16	
seed development			3
small molecule biosynthetic process		11	
UMP biosynthetic process		3	
< Cellular >			
chloroplast			13
COPI vesicle coat		4	
cytoplasmic part	46		
cytosol			11
cytosolic large ribosomal subunit		5	
DNA-directed RNA polymerase II, holoenzyme	4		
extracellular region	15	12	12
kinesin complex		3	
nuclear part		13	
organelle subcompartment			11
plasma membrane part		8	
transferase complex		9	
Molecular			
ATP-dependent microtubule motor activity		3	
IgE binding	3		3
inorganic diphosphatase activity	3		
metal ion binding	28	38	
nutrient reservoir activity	6		
oxidoreductase activity			25
peroxidase activity	6		
serine-type endopeptidase inhibitor activity			5
structural molecule activity		11	
uridine kinase activity		3	

### Grain and eating quality traits of 5MT R-lines

**Fig 5:**
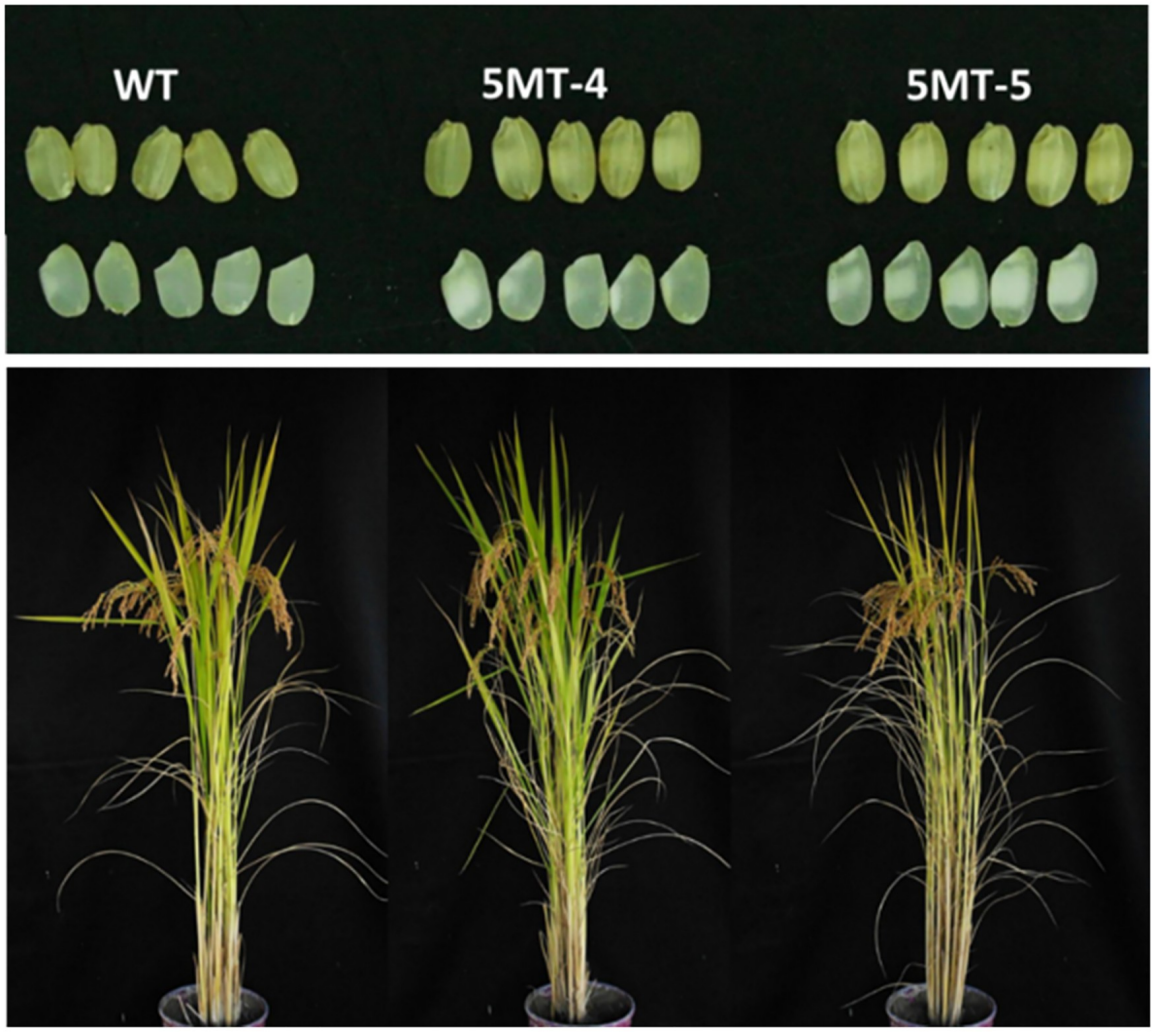
Grain phenotypes of unpolished (top row) and polished grains (bottom row) and phenotypes of WT, 5MT-4, and 5MT-5 in maturity stage from field condition.

Grain and eating quality tests in 5MT-4, 5MT-5 and WT were performed in this section. To obtain optimum results of grain and eating quality, it is very important to consider moisture content (MC) of grain after harvest. The optimum MC was approximately 12–14%. The average MC of rice materials used in the analysis fell in the range of 13–14%. Translucency and whiteness of WT was 82% which was higher than that of 5MT R-lines at 41–45% (Table 6). Actual grain phenotypes of WT, 5MT-4, and 5MT-5 in brown and milled rice forms are shown in Fig 5.

**Table 6:** Percentage of moisture content and whiteness, and grain quality traits in terms of whole grain, broken rice, chalky grain, and damaged grain of WT and 5MT R-lines.

Traits	WT	5MT-4	5MT-5
Moisture content	13.34 ± 0.02	14.37 ± 0.04	13.94 ± 0.01
Whiteness/Translucency	82.47 ± 0.14	45.23 ± 0.13	41.57 ± 0.29
Whole grain	87.87 ± 4.56^a^	51.93 ± 1.75^b^	34.33 ± 4.71^c^
Broken rice	5.70 ± 2.94^a^^b^	4.03 ± 0.67^b^	8.90 ± 2.60^a^
Chalky grain	6.07 ± 1.59^c^	43.43 ± 1.80^b^	55.60 ± 5.94^a^
Damaged grain	0.40 ± 0.10^a^	0.63 ± 0.40^a^	1.17 ± 0.49^a^

Each value is mean ± standard deviation.

^a,b,c^ Mean in the same row followed by the same letter are not significantly (p<0.05) different by Duncan’s multiple range test (DMRT).

To quantify other grain quality measurements, whole grain, brokenness, chalky grain, and damaged grain of milled rice were measured. Observations showed that these two high Trp rice lines had many damaged grains. They were very chalky. Whole grain percentage of WT was 88% which was higher than 5MT-4 at 52%. 5MT-5 had the least whole grain % at only 34% (Table 6). Broken rice % of WT was not significantly different from that of 5MT-4 or 5MT-5. In terms of chalky grain, 5MT-5 had the highest % at 56%. It was significantly higher than that in 5MT-4 at 43% and WT at 6%. Regarding the score of grain quality itself of polished milled 5MT R-lines, the score showed bad grain appearance. Chalkiness of grains was very high for 5MT-4 and 5MT-5 despite good harvesting and milling management.

### Protein and amylose contents in 5MT R-lines

Protein content of WT was 6.2%. It was significantly lower than the mean protein content of 5MT-4 and 5MT-5 at 7.2% (Table 7). The AC (%) of WT was not significantly different from that of mutants. WT and 5MT-5 had AC of 18% while 5MT-4 had AC of 19%. Dongjinchal, a glutinous rice, had AC as low as 3%.

**Table 7:** Protein, amylose contents, alkali digestion value and gelatinization temperature of WT, 5MT R-lines, and Dongjinchal, a kind of glutinous rice as control.

Traits (%)	WT	5MT-4	5MT-5	Dongjinchal
Protein content	6.19 ± 0.03^b^	7.19 ± 0.06^a^	12.94 ± 0.03^a^	7.1 ± 0.03^a^
Amylose content	18.10 ± 0.75^a^	19.13 ± 1.45^a^	18.42 ± 3.60^a^	4.07 ± 0.37^b^
Alkali digestion value (ADV)	4	6	6	2
Gelatinization temperature (°C)	70–74	55–69	55–69	75–79

^a,b^ Mean in the same row followed by the same letter are not significantly (*p* > 0.05) different by Duncan’s multiple range test (DMRT).

**Fig 6:**
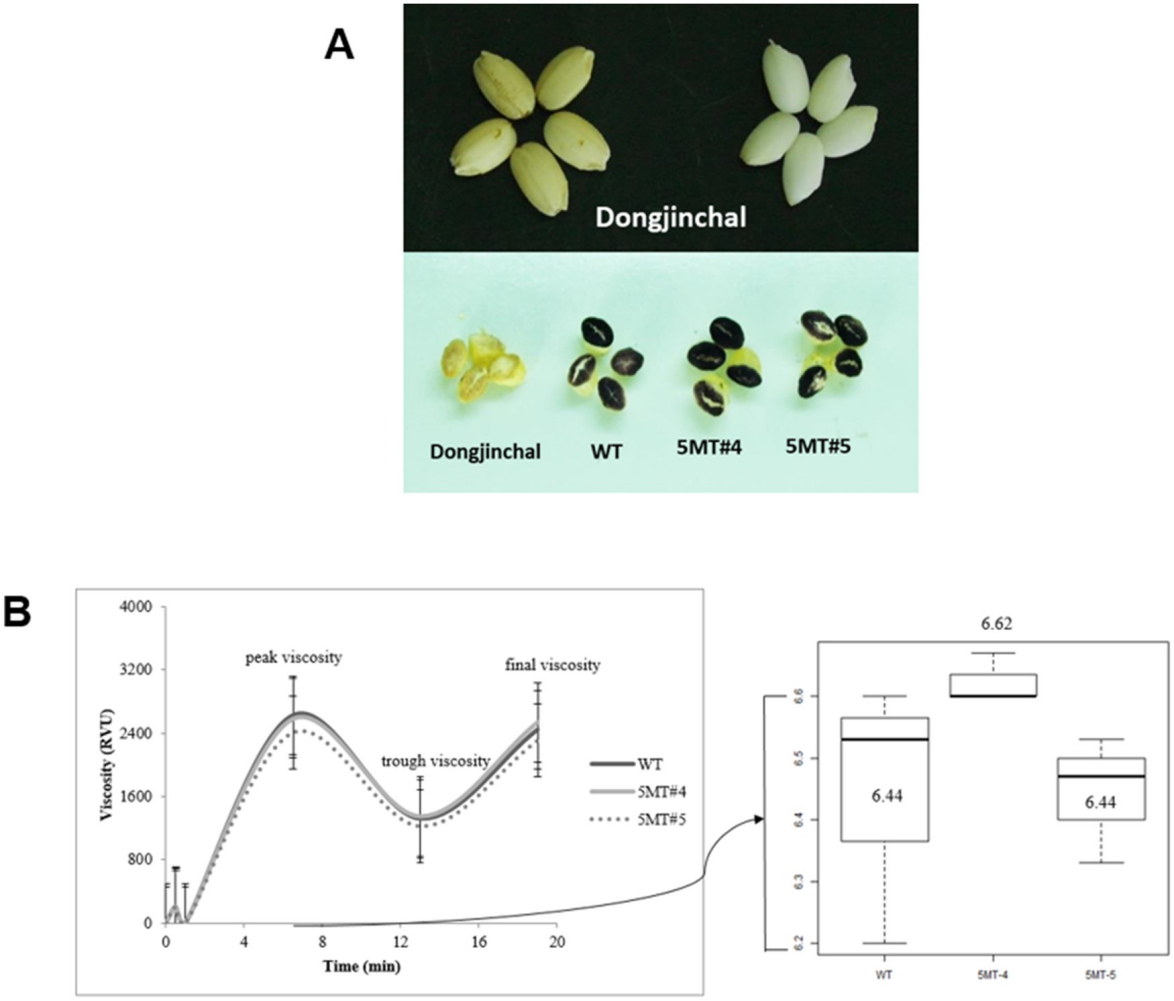
(A) Lugol’s test using cross sections of rice grains. Dongjinchal, a glutinous rice with low amylose content, served as a control. WT, 5MT-4, and 5MT-5 showed purple color which indicated the presence of high amylose content. (B) Viscosity curves obtained by RVA. Comparison of curves of the wildtype and two mutant rice lines with high levels of Trp.

A simple test for starch was performed using Lugol’s test. Dongjinchal was chosen as glutinous type for low starch control. Through visual scoring, it is easy to say which sample has a low or high amylose content because a blue-black color will show when starch is present. If starch amylose is not present, then the color will stay orange or yellow (Fig 6A). Each value is presented as mean ± standard deviation. Means in the same column followed by the same letter are not significantly (p > 0.05) different by Duncan’s multiple range test.

### Viscosity curves of 5MT R-lines

Fig 6B shows viscosity curves of WT and the two 5MT R-lines. Peak, trough, and final viscosities were the same for WT and 5MT-4 while 5MT-5 showed slightly lower viscosity. From the peak viscosity, peak time differences of the three samples were highlighted. The average peak time of WT and 5MT-5 was 6.44s while that of 5MT-4 was 6.62s. Results revealed that 5MT-4 had faster viscosity than WT and 5MT-5.

### Gelatinization temperature of 5MT R-lines

Based on the degree of alkali digestion observed, WT and the two mutants were visually scored. Alkali digestion values and corresponding gelatinization temperatures of samples are summarized in Table 7. 5MT-4 and 5MT-5 showed lower gelatinization temperature (55–69 °C) compared to WT (70–74 °C) whereas Donjinchal, a glutinous rice, showed the highest gelatinization temperature (75–79 °C).

### Taste scores of 5MT R-lines

Results of average taste parameters such as perception, stickiness, balance, and taste are shown in Table 8. The wildtype showed higher values than the two 5MT R-lines, although no statistical significance was found. Perception scores of the WT, 5MT-4, and 5MT-5 were 1.72, 1.56, and 1.03, respectively. In terms of hardness value, 5MT-5 showed the highest value at 8.92 compared to WT (8.57) and 5MT-4 (8.71). For stickiness value, WT had 2.27 which was higher than that of 5MT-4 or 5MT-5 (at 2.03 and 1.50, respectively). For balance, WT had the highest value (1.92) compared to 5MT-4 at 1.66 and 5MT-5 at 1.16. In terms of palatability score, values of WT, 5MT-4, and 5MT-5 were close to each other (70.5, 68.13, and 71.53, respectively).

**Table 8:** Eating quality traits of WT, 5MT-4, and 5MT-5.

Trait	WT	5MT-4	5MT-5
Perception	1.72^a^	1.56^a^	1.03^a^
Hardness	8.57^a^	8.71^a^	8.92^a^
Stickiness texture	2.27^a^	2.03^a^	1.50^a^
Balance	1.92^a^	1.66^a^	1.16^a^
Palatability	70.5^a^	68.13^a^	71.53^a^

^a^ Means in the same row followed by the same letter are not significantly (*p* > 0.05) different by Duncan’s multiple range test (DMRT).

## Discussion

Two mutant rice lines (5MT-4 and 5MT-5) that were resistant to a Trp analog, 5-methyl tryptophan, were characterized. Although many characterizations have been published, this is the first study to perform molecular characterization of OsASA^Δ^ gene and evaluation of grain and eating quality of 5MT R-lines. 5MT-4 and 5MT-5 were derived from seeds mutated with 2% EMS. Results showed that these two mutant lines were resistant to growth inhibition when grown in media having 50 μM 5MT [23]. This *in vitro* method is a good way to select rice seedlings resistant to 5MT. In addition, it has been reported that 5MT can inhibit the synthesis of anthranilate compounds known to be involved in the first steps of biosynthesis of Trp in *Neurospora crassa* [28]. 5MT is also a corepressor of *Escherichia coli* Trp repressor [29–30]. In normal feedback inhibition, once cells are exposed to Trp rich environment, signals to stop producing Trp will be produced in cells. However, due to mutations in anthranilate synthase gene, plants become insensitive and proceed to grow.

To further characterize these mutant lines, the *OsASA* gene of 5MT-4 and 5MT-5 was sequenced. Results confirmed that both lines had the same amino acid base change of F124V (TTC to GTC). In a previous study, other amino acid changes (S126F and L530D) in other 5MT R-lines have been reported [18], causing the same insensitivity of AS enzyme to feedback inhibition. AS enzyme activity assay was also performed for 5MT-4, 5MT-5, and WT. The activity of AS became more active (50-fold higher than normal) in 5MT-4 and 5MT-5 as the concentration of 5MT increased while the WT showed a decrease in AS activity. Thus, a single base change in *OsASA* that also changes amino acid base allows AS enzyme to have resistance to feedback inhibition in the presence of 5MT analog.

The gene expression level of DEGs identified from the microarray analysis is shown in S3 Fig. To narrow the gene expression analysis in terms of the whole Trp biosynthesis pathway, quantification of genes encoding key enzymes involved in the pathway was conducted. There are two alpha subunits of *OsASA* encoding the AS enzyme: AS1 and AS2. AS1 showed low expression at 1.75-fold in 5MT-4 and at 1.6-fold in 5MT-5 relative to the expression of WT with 2.25-fold change. AS2 showed high expression at 2.75-fold in 5MT-4 and low expression at 2.4-fold in 5MT-5. For enzymes APT, TSAC, and TSBC, the trend of their mRNA expression was the same as the WT when looking at the general pattern. However, mRNA expression levels of these genes were not equal to their WT. APT and TSAC had relatively lower while TSBC had higher expression levels in mutants compared to WT. In this pathway, AS converts chorismate to anthranilate, making it the key regulating enzyme.

The most considered trait in 5MT resistant mutants is the elevation of Trp content. In this study, Trp content was at least 20 times higher in 5MT-R lines than the WT for both brown rice and milled rice forms. Surprisingly, not only Trp content was elevated, but also contents of Asp, Glu, Asn, Ser, Gln, Gly, Thr, Ala, Val, Ile, and Leu were higher in 5MT-R lines than WT. In return, all these amino acids might have contributed to total amino acid contents in 5MT-4 and 5MT-5 that were significantly higher than WT. This is different from free amino acid composition results reported previously [10] because in their transgenic Azuki bean which is resistant to 5MT, only Trp content is increased in the seed. From this result, even after milling, Trp is still present in 5MT-resistant rice. Among 5-MT mutants, various genetic mutations may happen other than the key target compared to a transgenic-modified 5MT-resistant crop.

In this study, the transcriptome of five different time points (2, 5, 10, 15, and 20 DAP) of grain filling of wildtype Donganbyeo was assayed through microarray and compared with 5MT R-line of mutant rice. Results of microarray analysis between WT and 5MT R-line mutant rice reflected differences in transcriptome during grain filling of these two rice genotypes with significantly different total amino acid contents. Results at 2 DAP were omitted because gene expression values did not meet the 95% level of confidence. DEGs results showed that there were 399 DEGs (209-up, 190-down) at 5 DAP, 715 DEGs (450-up, 265-down) at 10 DAP, 616 DEGs (338-up, 278-down), and 1453 DEGs (605-up, 848-down) at 20 DAP. This implies an increasing frequency of DEGs as grain filling progresses. Also, expression level of DEGs between the two genotypes was high. Chromosome distribution analysis of DEGs showed consistent trend at all four time points. Chromosomes 1 and 3 were those with the highest number of DEGs while the rest of the chromosomes showed average frequency. To see overlapping and unique DEGs across four different time points, Venn diagram of up-regulated and down-regulated genes were generated (S2 Fig). Only a few DEGs were observed to be shared: 16 in up-regulation and 15 in down-regulation. Surprisingly, there were a lot of unique genes found in each time point. These findings were supported by gene expression level analysis results made through clustering (S3 Fig). Only a few DEGs were found in all four time points. They were not consistently expressed as up- or down- regulated. However, many of these genes were expressed in at least two time points with the same type of regulation.

Protein class categorization of DEGs in all four time points of grain filling was then performed. The top three protein classes that were observed from 5 DAP up to 20 DAP were hydrolase, oxidoreductase, and transferase. Interestingly, hydrolase, oxidoreductase, and transferase all seemed to work together to metabolize the use of N during grain filling which could account for the translocation of amino acids. Hydrolases are enzymes that catalyze the cleavage of a covalent bond using water. For example, esterase can break down ester bonds using water molecule. Oxidoreductases are enzymes that catalyze transfer of electrons from a donor (reductant) to an acceptor (oxidant) molecule generally using NADP (nicotinamide adenine dinucleotide phosphate) or NAD^+^ as cofactors. Lastly, transferases are enzymes that catalyze the transfer of a functional group from a donor molecule, often a coenzyme, to an acceptor molecule. All these enzymes encoded by the DEGs found in grain filling stages were involved in a catalytic pathway. Specific genes involved in transporter and transfer/carrier protein classes were enriched in the latter part. From the list of transporter class protein, 14 DEGs were up-regulated and 18 DEGs were down-regulated. While there were only few genes enlisted under transfer/carrier protein class, all of them were up-regulated except for only one gene.

To date, limited reports on 5MT R-lines have shown any deleterious phenotypic trait. Transgenic rice expressing OASA1D (carries mutation in *OsASA*) has been reported to show decreased spikelet fertility [31]. Here, despite the increase in Trp content, seeds of 5MT-4 and 5MT-5 were very chalky (~60%). Thus, their grains had low translucency. After milling, 5MT-resistant rice also showed high percentage of damaged grains. In terms of protein content, increased values were observed for 5MT-4 and 5MT-5 possibly due to increased total amino acid content. To check either there was an effect in eating quality and cooking quality, amylose content was measured. No significant difference was found in the obtained results. For viscosity, similar viscosity curves were observed for WT and mutants except for peak time of viscosity. 5MT-4 had higher peak time than WT while 5MT-5 showed lower peak time as shown in Fig 6B. This might be explained by different chalkiness of their grains.

For gelatinization temperature (GT), 5MT-4 and 5MT-5 had lower GT compared to WT whereas Donjinchal, a glutinous rice, had higher GT. This implies that the chalkiness in 5MT-4 and 5MT-5 could lower its GT. Finally, by using a rice taste analyzer, perception, hardness, stickiness texture, balance, and palatability scores of 5MT-R lines were found to be similar to those of WT. This study shows proof that expected and unexpected secondary changes might occur once a metabolic pathway like Trp biosynthesis is modified. These mutant lines generated are important sources for high Trp content although they have low grain quality. They might be useful for developing new high nutrient rice varieties in the future.

## Supporting information

S1 Table Primer sequences of genes related to Trp biosynthesis used for qRT-PCR. (DOCX)

S2 Table Summary of differentially expressed genes. (DOCX)

S1 Fig Chromosome distribution of DEGs in rice chromosomes in 5, 10, 15, 20 DAP. (TIF)

S2 Fig Overlapping and unique genes during the four different time points (5, 10, 15, 20 DAP) of grain filling. (TIF)

S3 Fig Gene expression levels at 5, 10, 15, 20 DAP of grain filling stage. (TIF)
