# Supplementary material for: Physico-chemical characterization and transcriptome analysis of 5-methyltryptophan resistant lines in rice
Source: PLoS One. 2019 Sep 18;14(9):e0222262. doi: 10.1371/journal.pone.0222262 (PMC6750609; doi:10.1371/journal.pone.0222262)
Supplement: S1 Table — (DOCX) [file pone.0222262.s001.docx]

**S1 Table.** **Primer sequences of genes related to Trp biosynthesis used for qRT-PCR**

| **Genes** | **Primer sequence (5’-3’)** |
| --- | --- |
| *OsASA1* Fw | ASTGGAGAGTTGSRTGATSATCT |
| *OsASA1* Rv | CTACGAAWGRTGRTTYAGCAAGA |
| *OsASA2* Fw | ATGGAGTCCATCGCCGCCGC |
| *OsASA2* Rv | CTATTCCTTGTCTACGAAAG |
| *OsAPT* Fw | CAAGATGGCTAAGGCAGCTCAG |
| *OsAPT* Rv | CAACTATTGGAAATCTTTATCCAG |
| *OsTSAC* Fw | GCCGGYGTMCAWGGTCTWRTRG |
| *OsTSAC* Rv | GCYCTSCCYATGATCACDCCA |
| *OsTSBC* Fw | TGGCTATGGTGTAGACACTGAC |
| *OsTSBC* Rv | CATCAAGGTACTTGCTGGCTGT |
| *Ubq10* Fw | CTGTTCTAGGGTTCACAAGTCTGC |
| *Ubq10* Rv | GGACACAATGATTAGGGATC |
