# Supplementary material for: Physico-chemical characterization and transcriptome analysis of 5-methyltryptophan resistant lines in rice
Source: PLoS One. 2019 Sep 18;14(9):e0222262. doi: 10.1371/journal.pone.0222262 (PMC6750609; doi:10.1371/journal.pone.0222262)
Supplement: S2 Table — (DOCX) [file pone.0222262.s002.docx]

**S2 Table.** Summary of differentially expressed genes

| Days after Pollination (DAP) | No. of up regulated DEGs | | No. of down regulated DEGs | Total | Total Affymetrix IDs | | Total  MSU IDs |
| --- | --- | --- | --- | --- | --- | --- | --- |
| 2-3 | | All *P*-values > 0.05 | | | | | |
| 4-5 | | 209 | 190 | 399 | 356 | 394 | |
| 7-10 | | 450 | 265 | 715 | 618 | 692 | |
| 12-15 | | 338 | 278 | 616 | 539 | 603 | |
| 20 | | 605 | 848 | 1453 | 1292 | 1433 | |
