# Supplementary figures and images for: Physico-chemical characterization and transcriptome analysis of 5-methyltryptophan resistant lines in rice
Source: PLoS One. 2019 Sep 18;14(9):e0222262. doi: 10.1371/journal.pone.0222262 (PMC6750609; doi:10.1371/journal.pone.0222262)

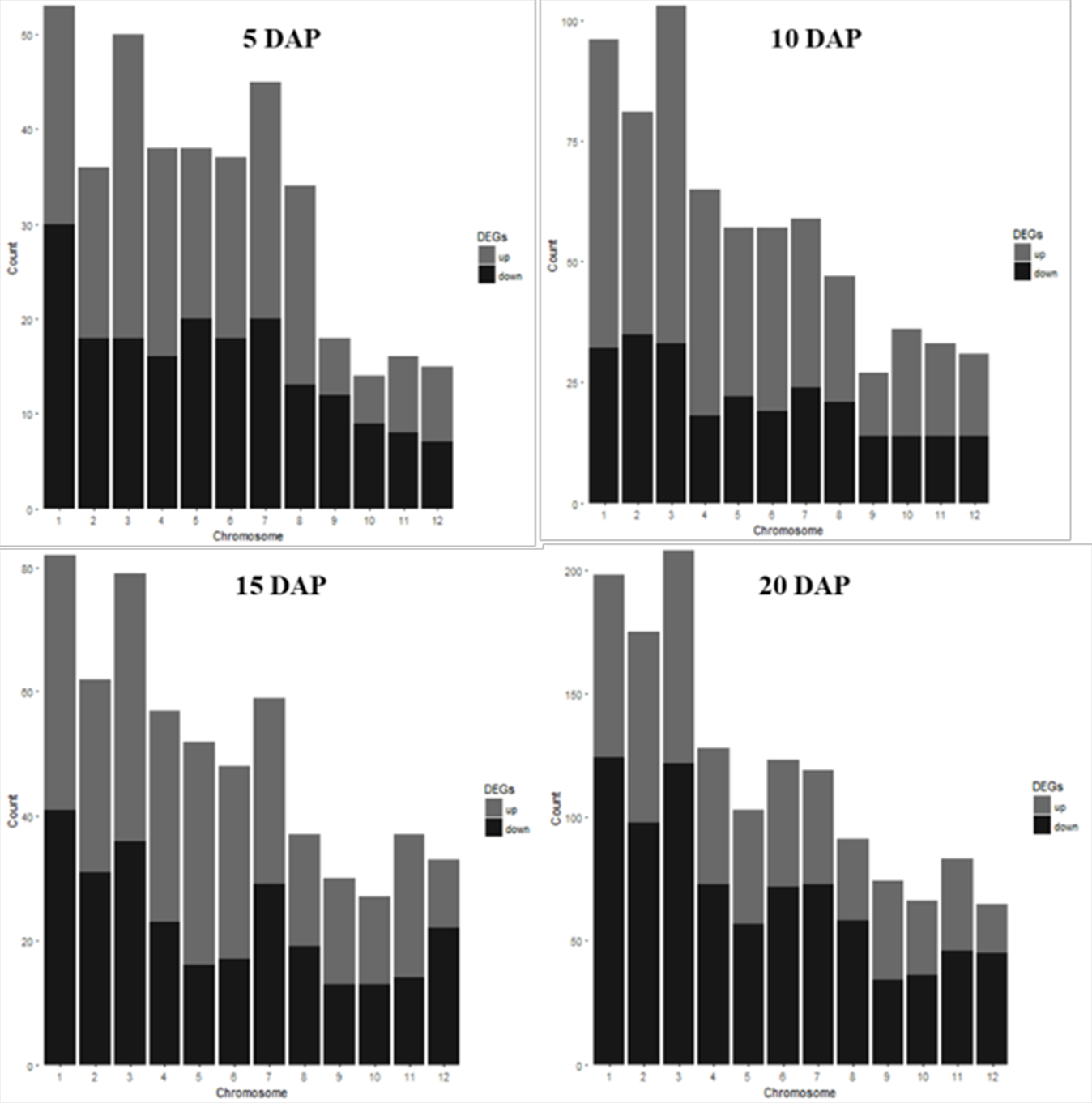

Supplement: S1 Fig — (TIF) [file pone.0222262.s003.tif]

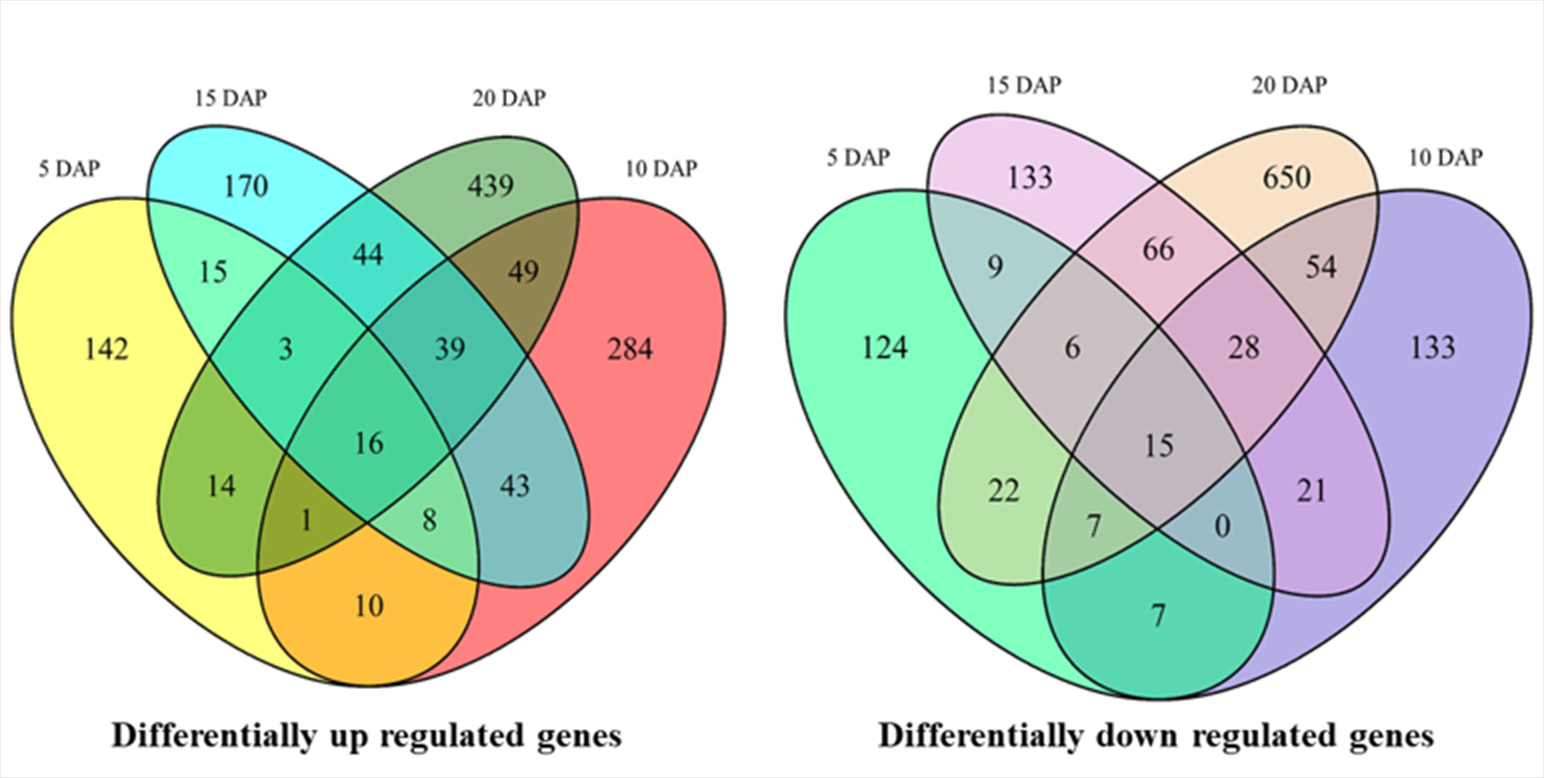

Supplement: S2 Fig — (TIF) [file pone.0222262.s004.tif]

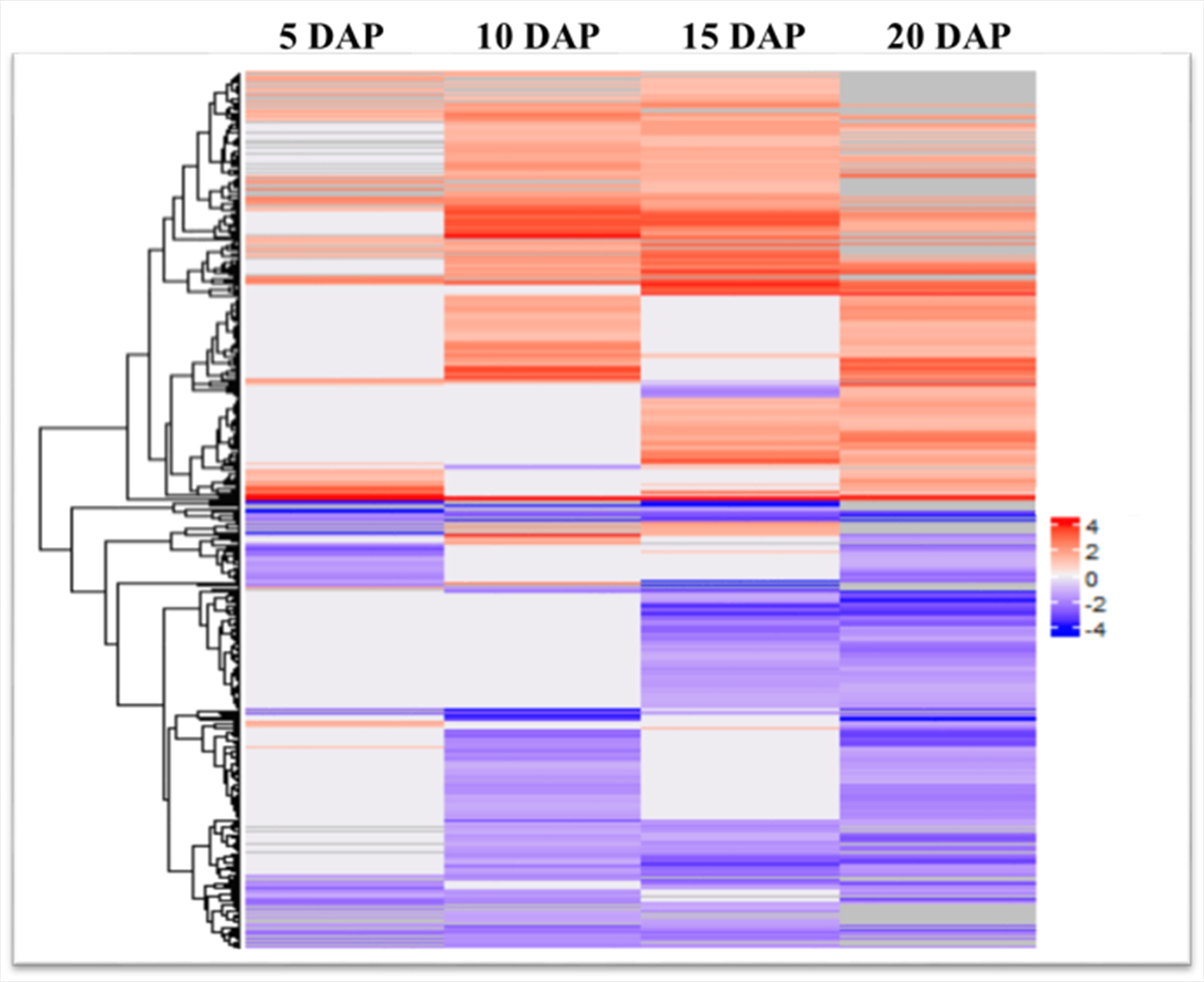

Supplement: S3 Fig — (TIF) [file pone.0222262.s005.tif]
